# Perspectives on Recruitment and Representativeness in Forensic Psychiatric Research

**DOI:** 10.3389/fpsyt.2021.647450

**Published:** 2021-06-17

**Authors:** Sven H. Pedersen, Henrik Bergman, Johan Berlin, Thomas Hartvigsson

**Affiliations:** ^1^Department of Psychiatry and Neurochemistry, Centre for Ethics, Law and Mental Health, Institute of Neuroscience and Physiology, Sahlgrenska Academy, University of Gothenburg, Gothenburg, Sweden; ^2^Forensic Psychiatric Clinic, Sahlgrenska University Hospital, Gothenburg, Sweden; ^3^Unit of Physiotherapy, Department of Health and Rehabilitation, Institute of Neuroscience and Physiology, Sahlgrenska Academy, University of Gothenburg, Gothenburg, Sweden; ^4^Lund Clinical Research on Externalizing and Developmental Psychopathology, Child and Adolescent Psychiatry, Department of Clinical Sciences, Lund University, Lund, Sweden; ^5^Department of Research and Development, Regional Forensic Psychiatric Clinic, Växjö, Sweden; ^6^Department of Philosophy, Linguistics and Theory of Science, University of Gothenburg, Gothenburg, Sweden; ^7^School of Philosophy and Art History, Faculty of Humanities, University of Essex, Colchester, United Kingdom

**Keywords:** forensic psychiatry, research participation, research ethics, representativeness, transparency, interpretability

## Abstract

Participant representativeness and statistical power are crucial elements of robust research with human participants, both of which relate to the successful recruitment of research participants. Nevertheless, such core features may often not be fully reported or duly considered in psychiatric research. Building on our experiences of collecting data in the context of forensic mental health services, we discuss issues regarding participant recruitment and representativeness in our field with its particular characteristics. A quick sampling and brief overview of the literature in four specialized forensic mental health journals is presented, demonstrating that published manuscripts rarely describe the data in sufficient detail for the reader to assess sample representativeness and statistical power. This lack of transparency leads not only to difficulties in interpreting the research; it also entails risks relating to the already meager evidence base of forensic mental health services being relevant only to a subset of patients. Accordingly, we provide suggestions for increased transparency in reporting and improved recruitment of research participants. We also discuss the balance of ethical considerations pertinent to the pursuit of increased participation rates in forensic mental health research.

## Introduction

Successful participant recruitment and sample representativeness are essential features in replicable and clinically applicable forensic psychiatric research. At the same time, experiences from our research group testify to difficulties in recruiting participants from forensic mental health services (FMHS) (1–3), with protracted recruitment periods and low participation rates as the norm. If this pattern is representative of FMHS research more broadly, as our discussions with colleagues from various countries suggest, it could constitute significant problems. Lack of high quality and representative, clinical research within the field of FMHS is problematic, both ethically [cf. (4)] and scientifically. However, applying more assertive recruitment strategies to this vulnerable group entails ethical challenges (5), highlighting the importance of recruitment strategies that are both effective and ethically sound. The aim of this paper is to investigate, discuss, and offer suggestions relating to the issues of recruitment, representativeness, and interpretability in FMHS research, while considering practical and ethical challenges.

Recruiting and retaining research participants is critical for clinical research in any field. Unsuccessful recruitment or high dropout rates may result in samples that are small, unrepresentative, or both. An insufficient number of participants limits the availability of methods of analysis, and the conclusions that can be legitimately drawn (6, 7). An unrepresentative sample has consequences for clinical applicability and generalizability. Some patient groups, potentially those most disabled (5), also run the risk of being systematically excluded by design (8) or by patients' unwillingness to participate (9). This could leave a subset of the population without the benefits that research could yield, such as service development tailored to their specific needs.

The FMHS setting entails particular challenges regarding sample size and representativeness. The overall population is comparatively small, meaning that low participation rates are likely to yield poor statistical power. The patient group is also highly heterogeneous, both within and between jurisdictions, in part due to legal criteria defining its limits. For example, in England and Wales offenders are transferred from prison to FMHS under the Mental Health Act if they have a mental disorder and pose a risk to either themselves or to others. In Germany and the Netherlands FMHS is regulated by the criminal code and patients are ordered by the criminal court to FMHS if they have a mental disorder and lack or have diminished responsibility for an offense. There are further differences between jurisdictions concerning what are considered relevant mental disorders for FMHS (10). This makes representativeness of samples a more challenging prospect. Although participant recruitment is an issue in medical research more broadly (11–15) the specific characteristics of FMHS may exacerbate such problems.

The risk of small or unrepresentative samples in FMHS research highlights the need for transparent reporting of recruitment-related factors in FMHS research. The reproducibility crisis (16–19) brought attention to this in the medical sciences in general, but the FMHS field has seen less scrutiny. Opaque reporting affects the interpretability of the research, as unrepresentativeness (20, 21) or inadvisable statistical practices (6) are not detectable.

If the opportunity to participate in research is an ethical right (4), it seems FMHS patients are seldom granted this right. Research pertaining to core interventions is lacking on a broad scale (22, 23). In an analysis of available systematic reviews, Howner et al. (22) concluded that none of the broad range of intervention categories they investigated had a sufficient evidence base in FMHS settings. Generalization from adjacent fields also appears problematic as grounds for service development, since FMHS differ in clinical practice (24, 25), patient treatment needs (26), and patient characteristics (27, 28). Length of compulsory inpatient care is also much longer for FMHS patients (29–31) than for patients in non-forensic services (32, 33). Furthermore, the care objectives in FMHS differ from adjacent service providers given its greater emphasis on the dual task of treating mental disorders and rehabilitating functioning as well as managing risks to others and to society at large (34, 35).

In striving to improve participation rates in FMHS research and to address the ethical risks and scientific shortcomings just described, other ethical risks emerge. One example is the risk of recruiting participants who do not or cannot give free and properly informed consent (5, 36). Informed consent in the FMHS context is problematic, even if adequate information is provided. In order for consent to carry ethical weight, a subject with *capacity to consent* must give it *voluntarily* (36, 37). First, a mental disorder can undermine capacity for consent (38–40). However, the mere presence of a serious mental disorder does not necessarily mean that a person lacks capacity to consent to research. The mental disorder needs to have a sufficient impact on the abilities underpinning capacity for it to undermine a person's capacity. Capacity to consent is decision-specific and may vary over time in one individual. Therefore, a person can have capacity to consent to some activities but not others (41).^1^ Second, FMHS is typically provided in an involuntary setting. Such a coercive setting risks exposing the patients to undue pressure to participate in research, especially if members of staff also carry out the research (43).

In sum, we suggest that there are challenges in trying to build a solid evidence base for FMHS due to difficulties in generalization from other fields, recruitment of research participants, and ethical challenges regarding informed consent. We also suggest that reporting practices need scrutinizing to assess risks of bias in the field. In the remaining sections of this paper, some of these challenges will be addressed. The section *Investigation into Transparency of Reporting Relating to Recruitment and Representativeness* presents a brief investigation into reporting practices and transparency regarding recruitment-related factors in FMHS journals. The section *Suggestions for Improved Reporting Practices* offers suggestions for reporting and research practices to increase transparency. In the section *Improving Recruitment Methods*, we offer suggestions to improve recruitment with due consideration of patients' autonomy.

## Investigation Into Transparency of Reporting Relating to Recruitment and Representativeness

To assess reporting practices and transparency in relation to participant recruitment, we collected a sample of studies published in FMHS journals and mapped the reporting of key recruitment-related variables (see Table 1 for specific variables).

**Table 1:** Numbers and percentages of the papers in our sample reporting power calculations, discussions of limits to representativeness in study, outline of sampled population, a priori selection of participants (inclusion and/or exclusion criteria at sufficient detail to assess the demarcation of the research population), dropout and/or refusal, explicit collection of informed consent, explicit mention of ethical review, discussions of ethics relating to recruitment or participation.

	**Power calculation**	**Discussion of representativeness**	**Outlines sampled population**	**A priori selection of participants**	**Dropout or refusal**	**Informed consent**	**Ethical review**	**Discussion on ethics of participation**
The Journal of Forensic Psychiatry and Psychology	0	4 (40%)	2 (20%)	6 (60%)	6 (60%)	8 (80%)	7 (70%)	3 (30%)
International Journal of Forensic Mental Health	3 (30%)	4 (40%)	4 (40%)	6 (60%)	4 (40%)	8 (80%)	9 (90%)	0
Criminal Behavior and Mental Health	1 (10%)	8 (80%)	6 (60%)	9 (90%)	7 (70%)	8 (80%)	10 (100%)	1 (10%)
International Journal of Law and Psychiatry	0	7 (70%)	5 (50%)	6 (60%)	5 (50%)	8 (80%)	9 (90%)	0
**Total**	4 (10%)	23 (57.5%)	17 (42.5%)	27 (67.5%)	22 (55%)	32 (80%)	35 (87.5%)	4 (10%)

For the investigation, we first composed a list of journals known to us to be operating in the intersection between criminal law and mental health. Second, the scope of each journal was inspected and those emphasizing research on law and psychiatry or mentally disordered offenders were retained. Third, the four^2^ journals with the highest impact factor^3^ were selected for analyses, yielding the following publications:

1. Criminal Behavior and Mental Health;
2. The Journal of Forensic Psychiatry and Psychology;
3. The International Journal of Forensic Mental Health;
4. The International Journal of Law and Psychiatry.

**Figure 1:**
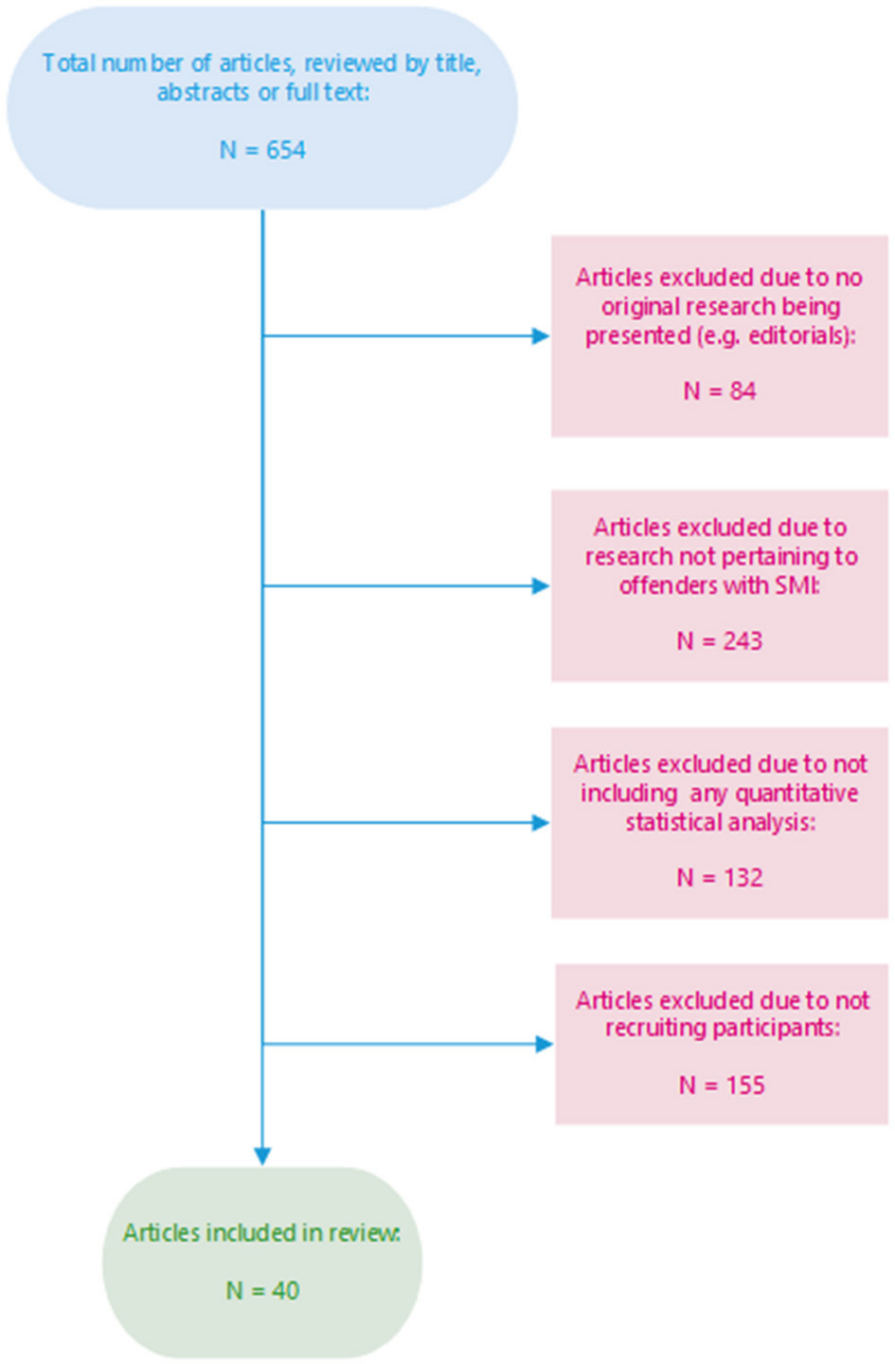
Overview of the article inclusion process.

Although relevant publications may have evaded our overview, we deemed the selection satisfactory to provide an initial sense of reporting transparency in specialized FMHS journals. For each journal, we selected the ten most recently published articles (see Supplementary Material 1 for complete list of included articles and assessment) which: (1) reported original research; (2) explicitly recruited participants from a population of mentally disordered offenders; and (3) employed inferential statistical analyses (see inclusion procedure in Figure 1). The papers were assessed based on whether they had employed a prospective power calculation, delineated the population from which the sample had been drawn, reported how the resulting sample was attained (e.g., inclusion/exclusion criteria, declining participation, dropout), reported whether the study had received ethical approval, reported collection of informed consent, or included any discussion on ethical considerations on recruitment or representativeness. Two authors independently reviewed each journal and article. Vague or ambiguous cases were discussed among all authors until consensus was reached.

Among the 40 papers included, 10% reported an a priori power calculation. Concerning representativeness, only a minority of papers presented the relation between the sample and the population in a manner that enabled assessments of the risk of bias. In 43% of papers, the authors provided the size and defined the boundaries of the total population from which the sample was drawn, 68% of studies reported inclusion and exclusion criteria in sufficient detail to assess exclusions related to study design, and 55% reported dropout frequencies or decline rates. 33% of the papers included sufficient data to trace how the sample was derived from the population it was intended to represent. Concerning informed consent, 80% reported collecting informed consent and 88% reported having received ethical approval by a review board. In addition, only 10% of papers included more than a cursory note on the implications of potential non-representativeness and systematic exclusion, or the ethics and utility of the presented research in light of this (see Table 1).

To summarize, a minority of papers presented results in a manner that enabled a thorough examination of the representativeness of the sample and the statistical power to detect a significant effect in that sample. In addition, few papers discussed the ethical or evidentiary implications of this. Such considerations and calculations may have been undertaken but not reported in some cases. However, to build a robust evidence base within FMHS transparent reporting is necessary, as opaque reporting risks concealing bias. Poor evidence may be worse than a known lack of evidence as a foundation for clinical practice.

## Suggestions for Improved Reporting Practices

Based on the investigation presented above and on our own experiences, we propose four strategies that can be employed by researchers and considered by editors and reviewers (in the remainder, we will discuss the practice of researchers, although the corresponding responsibilities for editors and reviewers should be kept in mind) to increase transparency and generalizability of FMHS research.

First, we suggest that researchers should be explicit in describing how their sample relates to the overall population it represents, including reports of inclusion and exclusion criteria, proportion of eligible participants who agreed to participate, and the attrition rate. Such reporting would enable the reader to better gauge the risk for bias in the sample [see (44, 45), for examples of clear reporting].

Second, we propose that non-participants and dropouts are described in as much detail as is ethically and practically possible. With ethical approval, existing clinical registers may provide anonymized demographic, clinical, and criminological data for the overall population, enabling rudimentary comparisons with the recruited sample [e.g., the National Forensic Psychiatric Register in Sweden (25)]. In longitudinal data collections, methods for managing dropouts, and the resulting missing data, with a reduced loss of information are available [e.g., (46, 47)] and can complement the above suggestion. If we fail to address these issues, the generalizability of our studies will suffer.

Third, relating to the accuracy of statistical analyses, we propose a routine application of prospective power analyses and sample size planning that take into account the difficulties of recruiting participants in the FMHS context. This would aid realistic appraisal of a project's viability and what conclusions can be drawn from a set of data.

Finally, we suggest a more widespread adoption of the practice of preregistration (16, 48). All of the suggestions above gain added weight if presented to the scientific community prior to data collection.

## Improving Recruitment Methods

### Collaboration Between Clinics and Researchers

Studies evaluating the effectiveness of recruitment practices are scarce (14, 15). The suggestions below are based on published articles that detail successful strategies for participant recruitment (49), identify barriers to recruitment (9, 11, 12, 15), and on a review of recruitment strategies (14). Close collaboration between researchers and clinics has been described as promising (11, 15). Early, ongoing engagement and communication between clinical staff and researchers is highlighted as a way to stimulate collaboration and recruitment (10). Additionally, the engagement of an intermediary (often clinical staff) who has an established relationship with the potential participant can help overcome barriers such as lack of familiarity with researchers, or to accommodate specific needs of an individual participant (9, 11).

Emphasizing the importance of collaboration, Sundeen et al. (49) reported on a model that may serve as an example. The model incorporated regular meetings between clinics and researchers as well as designated research liaisons at the clinics. It has yielded promising results concerning recruitment and reciprocal information sharing. Although not discussed in the published literature, efforts such as the involvement of service-user representatives^4^ or training clinical staff in research methodology may have a positive impact on collaboration and recruitment. For research activity to be sustainable over time at the clinic, it seems necessary that such connections are based on long term arrangements.

Incorporating research and clinical practice also carries risks, particularly when clinical staff act as intermediaries between researchers and participants. Researcher-independence, both in fact and in appearance, is crucial in coercive contexts such as FMHS (5). Beyond the risk of coerced consent, participants may be subject to the therapeutic misconception, conflating the goals of research with the goals of treatment [(50), see also (51, 52) who question this the gravity of this risk]. Similarly, and perhaps more pressingly in FMHS, participants' decision to participate or not may be influenced by how they expect such a decision to affect the clinical staff's perception of them and, consequently, their prospects for release (53). Participant information typically mentions the distinction between research and clinical practice, but these issues imply the need for very clear information on this point as well as a credible differentiation between research and care. Accordingly, the collection of informed consent ought to rest with a researcher who is markedly independent of the participant's care staff.

The sustainability of a continuous collaboration between participants, clinics, and researchers might also benefit from reciprocity concerning information (15). Even though participants and clinics provide data, published research reports are not always available or accessible to them. Communicating findings and conclusions to staff and patients in participating clinics could be motivated on an ethical basis but may also improve collaborations and future recruitment efforts.

### Rewards and Elimination of Obstacles

Offering financial rewards is a common and seemingly fruitful strategy for increasing participation rates (14, 54). Given the financial situation of most FMHS patients, it can be argued that such incentives may enact undue influence on their willingness to participate (55). However, other sources of influence likely overshadows this influence in the context of FMHS. Furthermore, monetary incentives are common among student samples and others of limited affluence and may even level out skewness in sample characteristics (56). Thus, monetary incentives seem defensible in FMHS research.

Facilitating participation and removing obstacles to participation is also encouraged (11, 15). Accommodating participants' logistical challenges, adapting to clinical schedules, and being flexible when an opportunity presents may help reduce attrition. These considerations are consistent with the views of persons who have declined research participation (9), although it is unclear whether those results translate directly to the FMHS context. Nudging participation by using an opt-out strategy for the provision of information may also aid recruitment (57). This only applies to the provision of information, as an opt-out strategy regarding consent seems inadvisable in a coercive setting.

### Multi-Site Collaboration

Given the generally low numbers of FMHS patients in any given location and the inevitable limitations to the suggestions above, multi-site and consortium-fueled research may be needed. Collaborative and distributed data collections serve to increase reach and sample sizes (and consequently power), as well as the representativeness across contexts and populations (58–61). Consistent application of widely used and available instrument enables pooling of data sets, which would facilitate collaborations across sites (62).

## Concluding Remarks

In light of the problems highlighted by the now well-known reproducibility crisis (16–19), the inherent heterogeneity of FMHS, and the overview presented above, we argue that the field is in need of increased transparency in the reporting of research. Good practice recommendations regarding research reporting can be borrowed from adjacent fields, where the discussion has been ongoing for some time. The area of participant recruitment is less developed and we hope that the issues and proposals presented above can contribute to increased attention to the matter. At present, we are part of a research programme of unprecedented scale in the Swedish FMHS context [FORevidence, (63, 64)]. The project's main goals are to (1) determine important areas for intervention, (2) to clarify the importance and implications of, and the preconditions for, user involvement, (3) to develop, adapt, and evaluate treatment methods for FMHS, and (4) to initiate a national platform for transdisciplinary forensic psychiatric research in Sweden. In this venture, we will strive to apply the suggested practices and to evaluate our recruitment strategies.

## Data Availability Statement

The original contributions presented in the study are included in the article/Supplementary Material, further inquiries can be directed to the corresponding author/s.

## Author Contributions

SP led the project, contributed to study design, literature review, and manuscript writing. HB, JB, and TH contributed to study design, literature review, and manuscript writing. All authors contributed to the article and approved the submitted version.

## Conflict of Interest

The authors declare that the research was conducted in the absence of any commercial or financial relationships that could be construed as a potential conflict of interest.

## Footnotes

^1^In the context of FMHS, capacity is also relevant in the issue of criminal responsibility. See (42) for a discussion on how conditions of criminal responsibility relate to conditions for capacity to consent.

^2^Choosing four journals was a pragmatic choice to split the work evenly between the four authors.

^3^Although Frontiers in Psychiatry – Forensic Psychiatry would have been included based on these criteria, we have opted not to. Two reasons motivated us to make this choice. The first is that a paper of this sort could risk unduly influencing the choice to publish as it could inflate the impact factor of the receiving journal if it is also included in the analysis. The second reason, which is related to the first, is that the journal's Editor in Chief is involved in this paper. Furthermore, after inspection of the published articles, three journals were excluded as research on mentally disordered offenders constituted a very limited share of published articles during 2019. These were Behavioral Sciences & the Law (3/50); Legal and Criminological Psychology (0/19); and Psychology, Crime & Law (1/54). The journals with the fifth and sixth highest impact factors were chosen to replace them.

^4^Concerning user-involvement, there is a risk of such arrangements becoming exploitative. User-involvement ought not to involve the performance of professional duties. As such, we consider such roles as the research liaison to be more suitable for a reimbursed employee than an unpaid service-user. Reimbursed user-representatives, such as can be the case in peer-supporter roles, would be less problematic in this sense.
